# A Novel Plenoptic Camera-Based Measurement System for the Investigation into Flight and Combustion Behavior of Refuse-Derived Fuel Particles

**DOI:** 10.1021/acsomega.2c08004

**Published:** 2023-05-01

**Authors:** Miao Zhang, Markus Vogelbacher, Krasimir Aleksandrov, Hans-Joachim Gehrmann, Dieter Stapf, Robin Streier, Siegmar Wirtz, Viktor Scherer, Jörg Matthes

**Affiliations:** † Institute for Automation and Applied Informatics, Karlsruhe Institute of Technology, 76131 Karlsruhe, Germany; ‡ Institute for Technical Chemistry, Karlsruhe Institute of Technology, 76131 Karlsruhe, Germany; § Department of Energy Plant Technology, Ruhr-University Bochum, 44801 Bochum, Germany

## Abstract

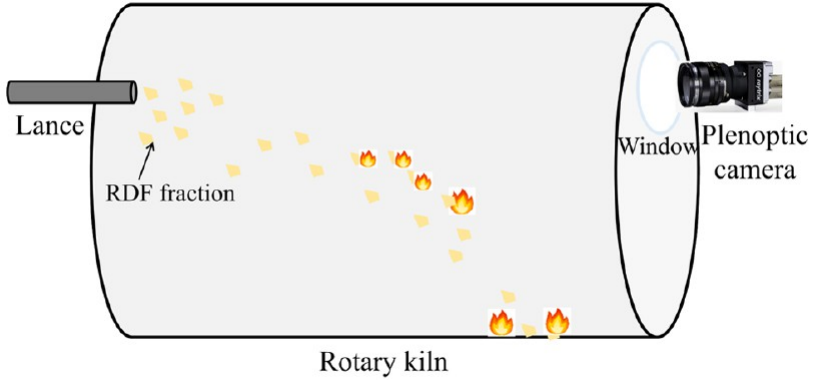

In the past several decades, refuse-derived fuels (RDFs) have been widely applied in industrial combustion processes, for instance, in cement production. Since RDF is composed of various waste fractions with complex shapes, its flight and combustion behaviors can be relatively complicated. In this paper, we present a novel plenoptic camera-based spatial measurement system that uses image processing approaches to determine the dwell time, the space-sliced velocity in the depth direction, and the ignition time of various applied RDF fractions based on the obtained images. The image processing approach follows the concept of tracking-by-detection and includes a novel combined detection method, a 2.5D multiple particle tracking algorithm, and a postprocessing framework to tackle the issues in the initial tracking results. The thereby obtained complete spatial fuel trajectories enable the analysis of the flight behaviors elaborated in the paper. The acquired particles’ properties (duration, velocity, and ignition time) reversely prove the availability and applicability of the developed measurement system. The adequacy and accuracy of the proposed novel measurement system are validated by the experiments of detecting and tracking burning and nonburning fuel particles in a rotary kiln. This new measurement system and the provided experimental results can benefit a better understanding of the RDF’s combustion for future research.

## Introduction

Recently, there has been a spate of interest in applying refuse-derived fuel in industrial combustion processes, for instance, in the cement production industry. Owing to the biogenic proportion of RDF, the utilization of RDF could benefit the CO_2_ balance in combustion processes. In addition, another significant merit of RDF compared to fossil fuels is the cost-efficiency. According to the study conducted by Weber et al.^1^ in 2015, which provides a comprehensive overview of waste treatment facilities in Germany, roughly 320 PJ of end energy are produced by waste treatment plants, including 225 PJ of heat and 90 PJ of electricity. RDF has been increasingly replacing fossil fuel, which positvely impacts the energy consumption. Typical RDFs are processed, solid, pneumatically conveyable fuels, which consist of various fractions, such as wood chips, plastic, and paper waste. Due to the complex composition of RDF, diverse investigations into its flight and combustion behaviors are entailed to ensure a controllable and effective application. Over the past several decades, researchers have contributed to studying the characteristics and behaviors of RDF. For instance, Duan et al.^2^ investigated the combustion behavior and pollutant emission characteristics of RDF in a pilot-scale vortexing fluidized bed combustor. Liedmann et al.^3^ presented a simplified modeling approach for analyzing the combustion and flight behavior of RDF processed from municipal or industrial waste based on an advanced fuel characterization starting with a sorting analysis of various fractions. The physical and thermal properties of extruded RDF are illustrated in ref (4) for the purpose of application in energy from waste technologies. Another comprehensive characterization of RDF in reference to the fuel technical properties is demonstrated in ref (5) where various examples are applied to highlight the influence of the properties on combustion behavior.

With the development of computer vision techniques, research of RDF’s properties utilizing image processing approaches has been widely undertaken. Pedersen et al.^6^ used an extraordinary camera setup that monitors combustion processes inside cement kilns to investigate cement plants with various kinds and percentages of alternative fuels. Krueger et al.^7^ demonstrated a novel experimental setup with a stereo camera system that aims to automatically determine the drag coefficient of particles with complex shapes, such as RDF particles. As an extension, Streier et al.^8^ conducted an investigation into the aerodynamic properties of RDF in a drop shaft by utilizing a stereo camera system. Vogelbacher^9^ carried out measurements for monitoring the combustion process of alternative fuel based on an infrared camera with a special spectral filter. In the study, the average flight distance of a conveyed bundle of fuel was also determined. In addition, a thorough review of versatile image processing techniques in fuel science is drawn up.^10^

The paper contributes to presenting a novel plenoptic camera-based measurement system aiming at investigating the spatial flight and combustion behaviors of diverse RDF particles. The collected data (captured images) from the camera are processed by image processing approaches of tracking-by-detection that tracks targets in accordance with previous detections. Based on the obtained spatial trajectories of the RDF fuel particles, we are able to discuss several flight and combustion properties. Additionally, the acquired outcomes give statements concerning the validity and feasibility of the developed measurement system reversely.

The principle idea of tracking-by-detection consists of two essential components: detection and tracking. Object detection is a vital task in image processing and has been extensively studied in various fields to identify diverse objects from macroscopic to microscopic in size. Our work focuses on detecting RDF particles without specific appearance properties. Principally, detecting these kinds of objects, such as cells and nuclei, is regarded as particle detection. Irshad et al.^11^ and Nicholson and Glaeser^12^ conducted detailed reviews concerning approaches to detect nuclei and electrons, respectively. Because of the utilized plenoptic camera that enables a spatial measurement with a single camera, scenes are able to be captured in 2D gray value images and 3D point clouds. In the present work, we propose a novel combined detection approach that takes both the gray value information and 3D point clouds into consideration. The developed approach identifies particles by combining and validating the outcomes of two utterly different detection principles: gray value based Scale Invariant Feature Transform (SIFT)^13^ and Density-Based Spatial Clustering of Applications with Noise (DBSCAN) clustering.^14^ The approach is initially introduced in ref (15) and proved sufficient accuracy and adequacy.

The particle detections ought to be associated temporally afterward to build complete particle trajectories, which are also the result of an object tracking approach. In order to track multiple particles, we recommend a 2.5D multiple particle tracking method based on the linear Kalman filter^16^ and the Global Nearest Neighbor algorithm (GNN)^17^ for state prediction, estimation, and data association. Principally, the particles can be tracked spatially. Nevertheless, the camera delivers depth information with considerable fluctuations that limit the possibility of direct 3D tracking. Thus, we introduce the 2.5D tracking by considering the depth information as a gating factor. Additionally, we also invent a postprocessing framework to deal with false detections and tracklets. Subsequently, the acquired 2D particle trajectories are converted into 3D trajectories whose fluctuations are then compensated by polynomial regressions.

Because of the combined detection approach, the 3D trajectories contain temporal particle positions and average gray values, on the basis of which we are able to analyze the RDF particles’ flight and combustion behaviors, including dwell time, velocity, and ignition time. These behaviors are of crucial significance and could instruct RDF applications in several cases. The acuqired fuel properties are compared with a CFD model, which shows a high agreement with the experimental results. Under the circumstances, the validity and availability of the proposed novel measurement system can be proved.

**Figure 1:**
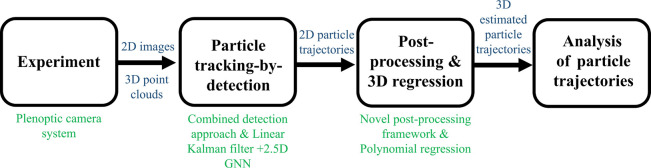
Schematic overview of investigation into flight and combustion behaviors of refuse-derived fuel particles based on a plenoptic camera system.

The diagram in Figure 1 outlines the entire work. In the Experiment Setup section, we elaborate on the test facility BRENDA (German abbreviation of Brennkammer mit Dampfkessel, in English, combustion chamber with steam boiler) located at the Karlsruhe Institute of Technology (KIT) Campus North and the utilized plenoptic camera system. The Image Processing Methods for Tracking-by-Detection section introduces briefly the image processing approaches of particle tracking-by-detection that contain a novel detection approach, a 2.5D multiple particle tracking method, a postprocessing framework, and a polynomial estimation of the initial fluctuated 3D trajectories. In the Results of Image Processing-Based Tracking-by-Detection section, we present the results of tracking-by-detection. For the sake of quantitative validation of the algorithms, the 2D particle positions and trajectories from several frames are manually labeled as ground truth. Following the results from the Results of Image Processing-Based Tracking-by-Detection section, the Discussion of Flight and Combustion Behaviors of RDF Particles section discusses the mentioned flight and combustion behaviors of the RDF particles.

## Experiment Setup

**Figure 2:**
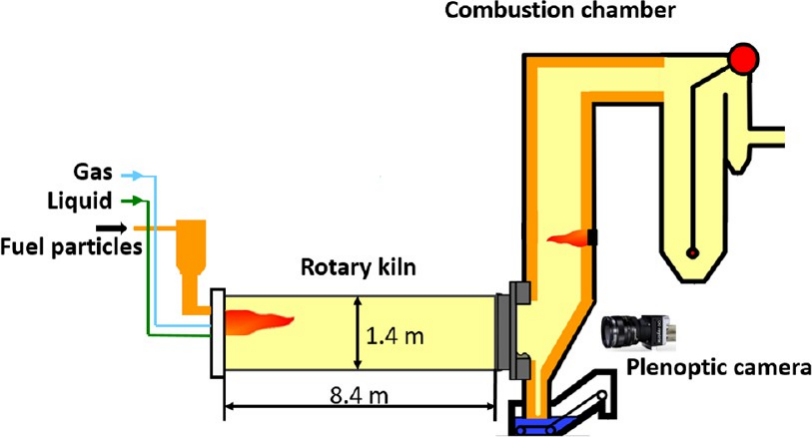
Schematic of the rotary kiln and the postcombustion chamber of the test facility BRENDA. Reprinted in part with permission from ref (18). Copyright 2019 Chemosphere.

The test facility BRENDA is a power plant combustion chamber on the pilot scale with a rotary kiln and a cylindrical, vertically arranged combustion chamber with a thermal output of 2.5 MW. BRENDA primarily comprises a rotary kiln and a postcombustion chamber that could provide a thermal power of 1.5 MW and 1 MW, respectively.^18^ Moreover, BRENDA is equipped with a boiler for heat recovery and a flue gas cleaning system. The test facility allows scalable experiments under conditions comparable to real cement industries. In the present work, the experiments are conducted in the rotary kiln that rotates with an angular velocity of 0.2 rpm. The rotary kiln rotates with the minimum speed to avoid sticking particles to the rotary kiln. As schematically depicted in Figure 2, the rotary kiln has a length of 8.4 m and an inner diameter of 1.4 m. Various RDF fractions are manually conveyed into the rotary kiln through the lance under the air feed pressure varying from 0.5 bar (outlet velocity ∼4 m/s, volume flow 70.5 m^3^/h) to 5 bar (outlet velocity ∼16 m/s, volume flow 76.6 m^3^/h). The conducted experiment primarily experiments with air feed pressures of 4.5 and 5 bar since the particles get stuck inside the lance with a pressure of less than 4 bar. The inside temperature of the rotary kiln is increased by the oil burner and could reach a maximum of 1240 °C limited by the technical conditions. Therefore, several fractions might ignite during their flights with different ignition times.

**Figure 3:**
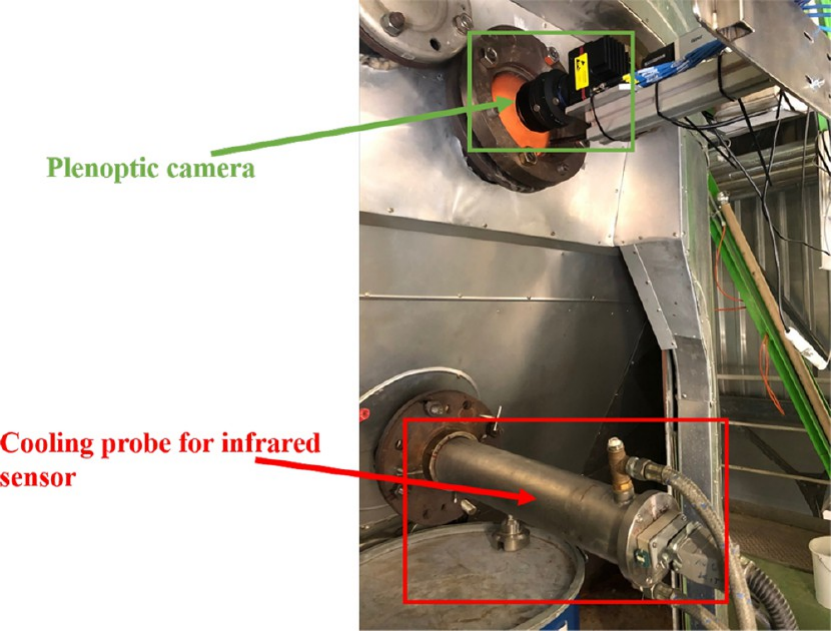
Images of the experiments conducted in the rotary kiln. The plenoptic camera mounted outside the kiln marked with a green rectangle is triggered by a computer. An infrared camera cooled by a cooling probe is marked with a red rectangle.

The particles are monitored by a high-speed plenoptic camera mounted outside the rotary kiln, as shown in Figure 3. The axis of the plenoptic camera coincides with the kiln’s rotation axis. The camera is fixed on a traverse in front of the glass window. Meanwhile, a computer on the right-hand side of the rotary kiln triggers the camera. The R12 plenoptic camera produced by the company Raytrix located in Kiel, Germany, provides a lateral resolution of 3072 pixels × 4096 pixels and a depth resolution of 1536 pixels × 2048 pixels. The used plenoptic camera is the Plenoptic Camera 2.0 (focused plenoptic camera) with a microlens array of three distinct focal lengths. This multifocus plenoptic camera enables a deep depth of field and a high maximal lateral resolution.^19^ The plenoptic camera captures images with a framerate of 330 frames per second (fps). According to the study of Sandemann,^20^ the accuracy of the depth information recorded by the plenoptic camera is facilitated significantly by a big lateral resolution, a big focal length, and a short distance between sensor and object. Hence, we select the camera with a focal length of 85 mm.

In order to record the spatial motions of RDF particles, a camera system capable of performing 3D measurements is essential. 3D cameras, such as stereo camera systems, time-of-flight (ToF) cameras, structured light RGB-D cameras, and plenoptic cameras, have been widely applied in technical and industrial measurements. Compared to other 3D cameras, the plenoptic camera could deliver sufficient accuracy and resolution when measuring small particles at long distances (2–8 m). Meanwhile, a plenoptic camera entails only a single aperture and trigger in measurements. Therefore, we give priority to a plenoptic camera system in the undertaken research.

**Figure 4:**
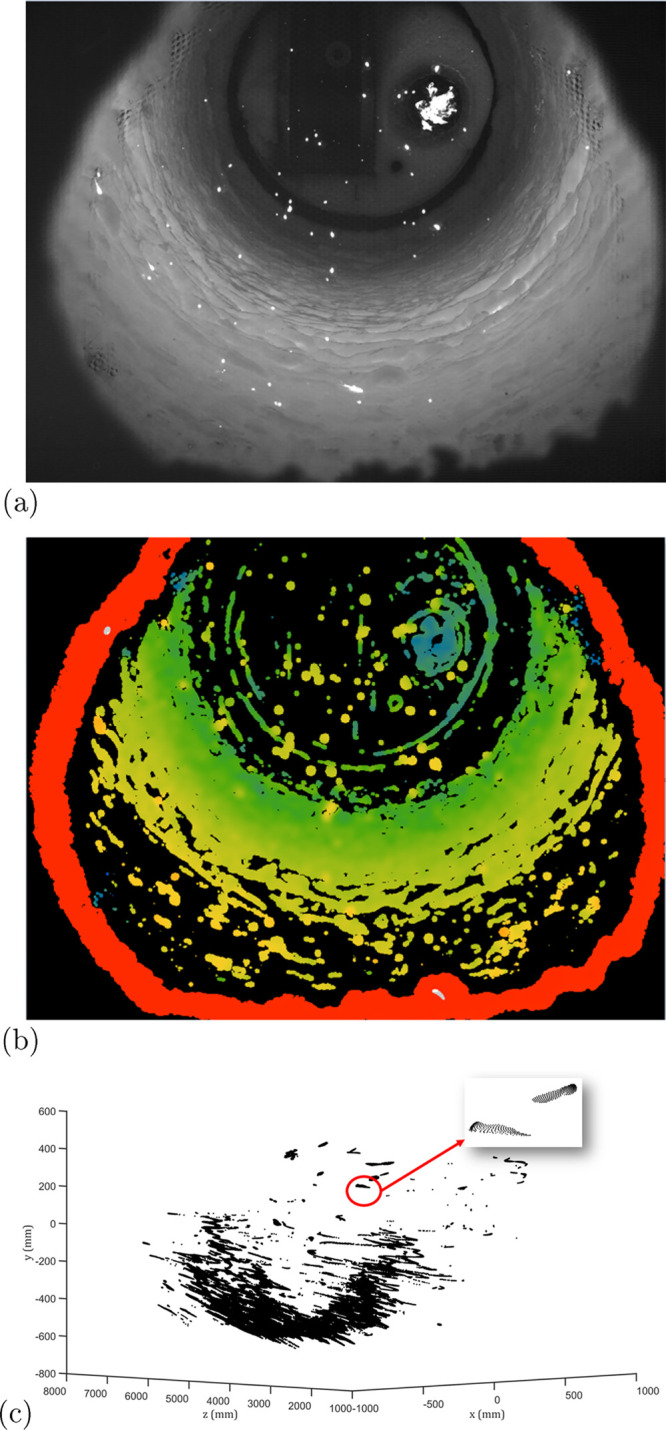
Examples of captured image in the rotary kiln. (a) Total focus image. The captured image corresponds to the image captured by a conventional camera. (b) Depth map. The depth information is depicted as a false-color image. Black indicates no depth information available. (c) 3D point cloud.

As a consequence of the plenoptic camera, the image information is available in both 2D gray value and 3D point cloud, which further benefits the image processing. Examples of the captured images are depicted in Figure 4. Figure 4a is the gray value image captured as a conventional camera. Figure 4b is the corresponding pixel-by-pixel depth map in false color, where black indicates no depth information available. In addition the depth information, the other two spatial coordinates of a pixel are stored as well. Figure 4c is a converted point cloud, where each pixel owns a specific spatial position with *x*, *y*, and *z* coordinates. Principally, the total amount of points in the point cloud could reach the depth resolution (1536 × 2048). Whereas not every pixel can be captured three-dimensionally, as shown in Figure 4b, the actual point cloud contains much fewer points than the maximum.

**Figure 5:**
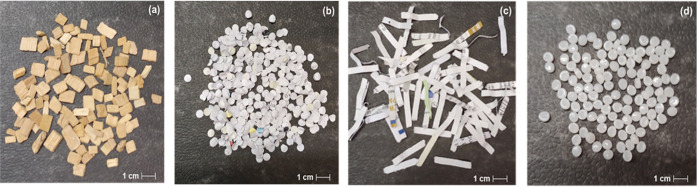
Various RDF fractions applied in the experiments. (a) Wood chips. (b) Confetti. (c) Paper shreds. (d) PE granules.

RDF contains a broad range of fractions, and we select four representative fractions among them, namely, wood chips, confetti, paper shreds, and polyethylene (PE) granules, as shown in Figure 5. The wood chips are premium smoking chips provided by the company JRS group (Rosenberg, Germany), while the PE granules are produced by the company Lyondelbassel (Rotterdam, The Netherlands). The particles’ actual sizes can be roughly estimated according to the 1 cm scale at the bottom right of Figure 5. Table 1 lists the 3D dimensions and the calorific values of the fuel fractions. In addition to the depicted four fractions, we also experimented with plastic foils. Nevertheless, the plastic foils are slightly visible due to their transparency despite an intense illumination, which constrains the possibility of image processing. Furthermore, plastic foils combust with considerable smoke that gathers in front of the camera and covers the particles, which dramatically challenges the difficulty of image processing. The outcomes of the experiments with plastic foils fail to provide appropriate basics for particle tracking-by-detection. Therefore, the paper presents flight and combustion behaviors concerning the depicted four fractions.

**Table 1:** Physical and Chemical Properties of Each Experimented Fraction (Calorific Value of Various RDF Fractions According to Refs (21) and (22))

	wood chip	confetti	paper shred	PE granule
form	cuboid	round flake	long flake	round plate
length (mm)	5–10	6	25–35	4
width (mm)	4–7	6	6	4
thickness (mm)	1	0.104	0.104	2
calorific value (MJ kg^–1^)	14.4	13.77	13.77	46.2

## Image Processing Methods for Tracking-by-Detection

As mentioned in the first section, the original and foundational idea of the paper is the analysis of RDF flight and combustion properties based on the obtained particle trajectories by means of image processing methods for tracking-by-detection. In this section, the particular approaches to detecting and tracking particles are demonstrated.

### Multiple Particle Detection

**Figure 6:**
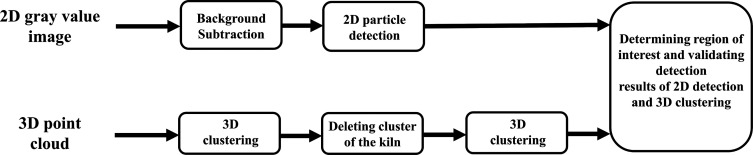
Schematic of the novel combined detection approach.

The novel combined detection approach compares and validates the detection results of the 2D gray value based detection method SIFT and the 3D clustering algorithm DBSCAN. The basic underlying principle of the approach is schematically illustrated in Figure 6. At first, SIFT and DBSCAN are implemented separately to generate two independent results. To address the issue of detection errors caused by the rotary kiln in the background, the 2D background model ought to be built and subtracted from the original images before SIFT detection. In the present work, we simulate the background by computing the temporal median gray value pixel-by-pixel within a certain relevant time period. We have studied and experimented with various background subtraction algorithms reviewed in refs (23) and (24), e.g., the frame difference approach, the adaptive background learning, and the running Gaussian average. The utilized median background subtraction algorithm could achieve satisfactory accuracy with a low computational cost. In 2D detection not only does the rotary kiln exert a negative effect on detection performance, but points belonging to the rotary kiln could also raise the number of false-positive detections in 3D clustering. Hence, we delete the cluster of the rotary kiln after the first clustering procedure and consider the outcome of the second clustering with distinct parameter values as the final clustering result. Particular problems persist in both 2D-SIFT and 3D-DBSCAN. For instance, SIFT fails to identify small particles with slight brightness, whereas 3D-DBSCAN could identify one large particle as several small particles due to unfavorable point distributions on occasion. Therefore, we decided to combine the detection results for the purpose of complete utilization of the delivered image information (2D gray value and 3D point clouds) to enhance detection accuracy. In the case of different detection results of a certain particle, the defined particle area will be marked, in which the gray value distribution will then be analyzed extensively for the definitive decision of the particle detection.

### Multiple Particle Tracking and Postprocessing

**Figure 7:**
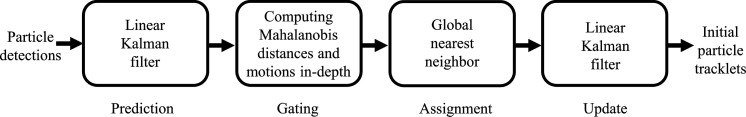
Schematic process of the multiple particle tracking.

The acquired temporal particle detections serve as 2D multiple particle tracking input. Generally, the tracking process comprises four steps: prediction, gating, assignment, and update, as shown in the flowchart in Figure 7. At first, the linear Kalman filter predicts the current particle positions and their corresponding covariance matrixes based on the existing tracklets. In the light of the high-speed plenoptic camera at 330 fps, the motions of the particles within two consecutive images can be regarded as uniform movement. In accordance with the predefined gating Mahalanobis distance, each prediction owns a gating ellipse under the consideration of the covariance matrix. In the present work, we restrict the gating process by introducing the particle movements in depth as a second gating factor. As rational assignment candidates of a certain track, their motions in the depth direction must be within a reasonable range. After determining assignment candidates of all tracks, their assignment costs are computed by the algorithm presented in ref (17), which build the cost matrix, subsequently. The final assignment decision is made by computing the minimal global cost, which corresponds to the global nearest neighbor approach. Afterward, the tracks are updated by the linear Kalman filter based on the assignments.

**Figure 8:**
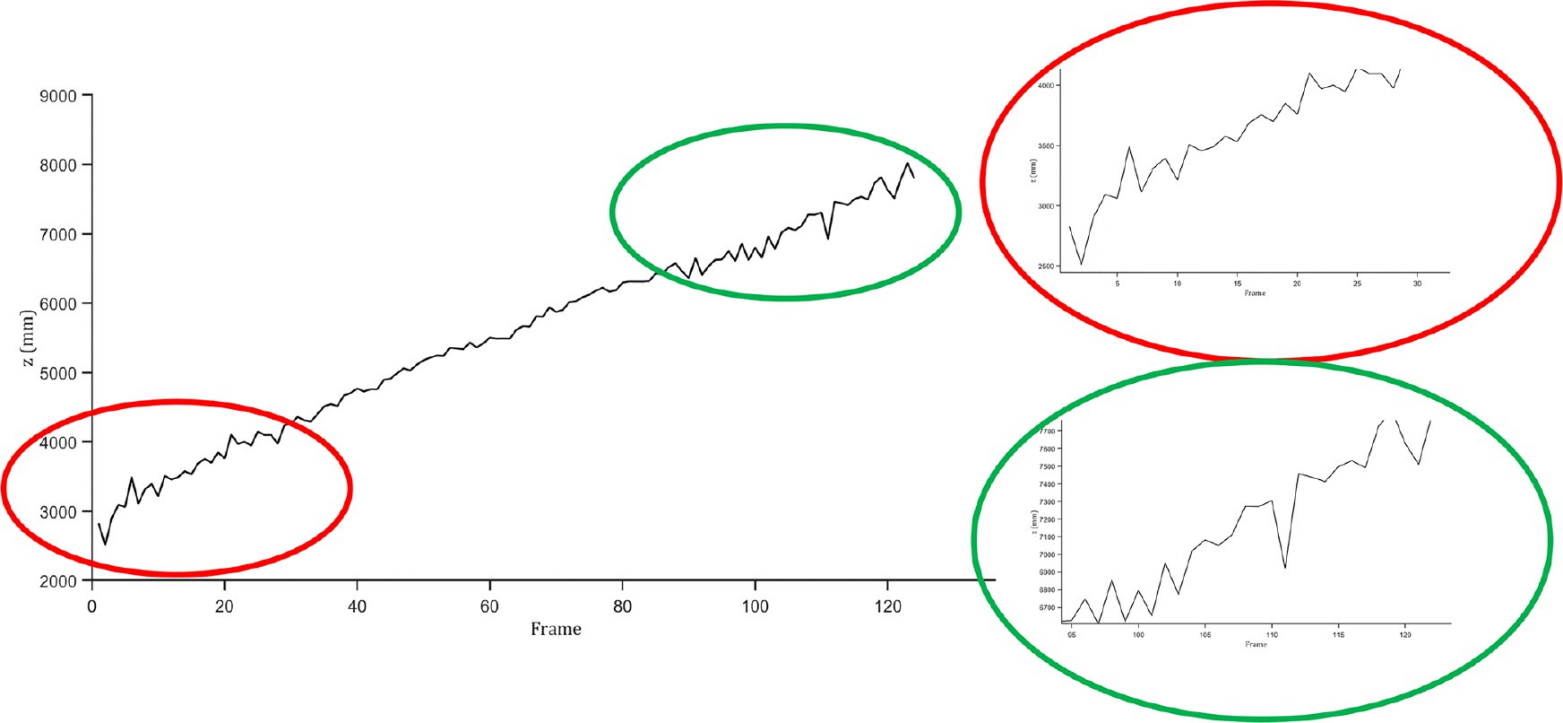
An example of particle trajectory. Two parts of the trajecory are zoomed-in to highlight the fluctuations inside the trajectory.

Principally, the demonstrated tracking approach is able to track particles spatially. Nevertheless, the plenoptic camera is not capable of providing steady depth information with acceptable fluctuations, as depicted in Figure 8. The fluctuated depth (up to 800 mm per adjacent frame) impacts the tracking performance dramatically since the motions in-depth are significantly larger than the motions in the other two directions. Thus, we track the particle two-dimensionally in image coordinates and regard the motion in-depth as a gating factor.

**Figure 9:**
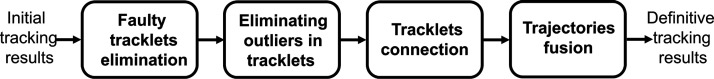
Postprocessing framework.

To deal with initial tracking results in the presence of faulty tracklets caused by detection errors (miss detections, duplications, or noise), we developed a postprocessing framework comprising false tracklets elimination, deleting outliers in tracklets, tracklet connection, and trajectory fusion. The entire postprocessing is schematically illustrated in Figure 9. At first, the tracklets that cannot fulfill a set of requirements, e.g., length and arithmetic average velocity, are eliminated. Subsequently, the detections in each tracklet are verified, and the checked outliers are deleted. With the completion of these two preparation steps, the remaining tracklets are connected by applying a proposed dual nearest neighbor approach detailed in refs (25) and (26). At last, we examine the connected trajectories and eventually fuse several trajectories in case they represent an identical particle’s trace.

The outcomes of the postprocessing framework are the definitive 2D particle trajectories in image coordinates. These trajectories are converted into 3D in accordance with the pixel-by-pixel coordinate information provided by the camera. Since the initial spatial trajectories fluctuated significantly, as shown in Figure 8, the trajectories should be smoothed for the purpose of a further statistical investigation of the flight-and combustion behaviors of the particles. The trajectories are estimated by third-degree polynomials independent in *x*, *y*, and *z* coordinates under the condition that all trajectories must start from the lance, which is regarded as a specific spatial point. In terms of this condition, the three polynomials ought to pass the lance position simultaneously and, thus, should be estimated jointly. This allows us to determine when a particle leaves the lance, even if the particle can only be detected later during the flight. In the present work, the polynomial regression is implemented by solving least-squares employing the trust region algorithm.^27^

## Results of Image Processing-Based Tracking-by-Detection

To realize a quantitative and objective validation of particle tracking-by-detection results, we manually count and follow particles in the images at first and consider the thereby obtained temporal particle positions and traces as ground truth for detection and tracking, respectively. In the following, the performance of the multiple particle detection and tracking are detailed.

### Results of Multiple Particle Detection

As ground truth, we selected 50 inconsecutive images with distinct particle distributions and labeled the particles regardless of their fractions in these images. By choosing different images with the various amount and percentages of burning particles, we aim to achieve a comprehensive and accurate evaluation of the detection performance. With regard to an undetermined threshold value, which affects the performance of 2D-SIFT considerably, we decided to select the value by using the resampling method cross-validation. The 50 ground truth images are divided into five groups in accordance with the captured time, with four groups as the training data set and the rest as the testing set each time. The optimal threshold value for the training data set is computed and regarded as the threshold for the testing set. Consequently, particle detection is processed. The 50 images’ detection results are summarized and compared with the ground truth. By virtue of the developed evaluation framework utilizing the Kuhn–Munkres algorithm,^28^ the detections are matched with the ground truth particles. Hereby, we denote the successfully matched detections as true positive (TP) and those without corresponding ground truth particles as false positive (FP). Misdetected particles in ground truth refer to a false negative (FN). Consequently, the precision, recall, and F1 score are computed as follows:
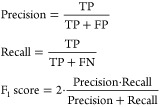
Table 2 gives a vivid overview of the detection performance of 2D-SIFT, 3D-DBSCAN, and the novel combined approach. Generally, 2D-SIFT performs more precisely than 3D-clustering, especially in terms of measurement precision, since several parts of the dark rotary kiln could be captured spatially as well. The proposed combined approach provides the best results regarding the three listed measurements.

**Table 2:** Performance of Particle Detection via 2D-SIFT with Median Background Subtraction, 3D-DBSCAN Clustering, and the Novel Combined Approach

	precision	recall	F_1_ score
2D-SIFT	0.9124	0.8404	0.8749
3D-clustering	0.4167	0.7237	0.5289
combined approach	0.9431	0.8655	0.9026

### Results of Multiple Particle Tracking

**Figure 10:**
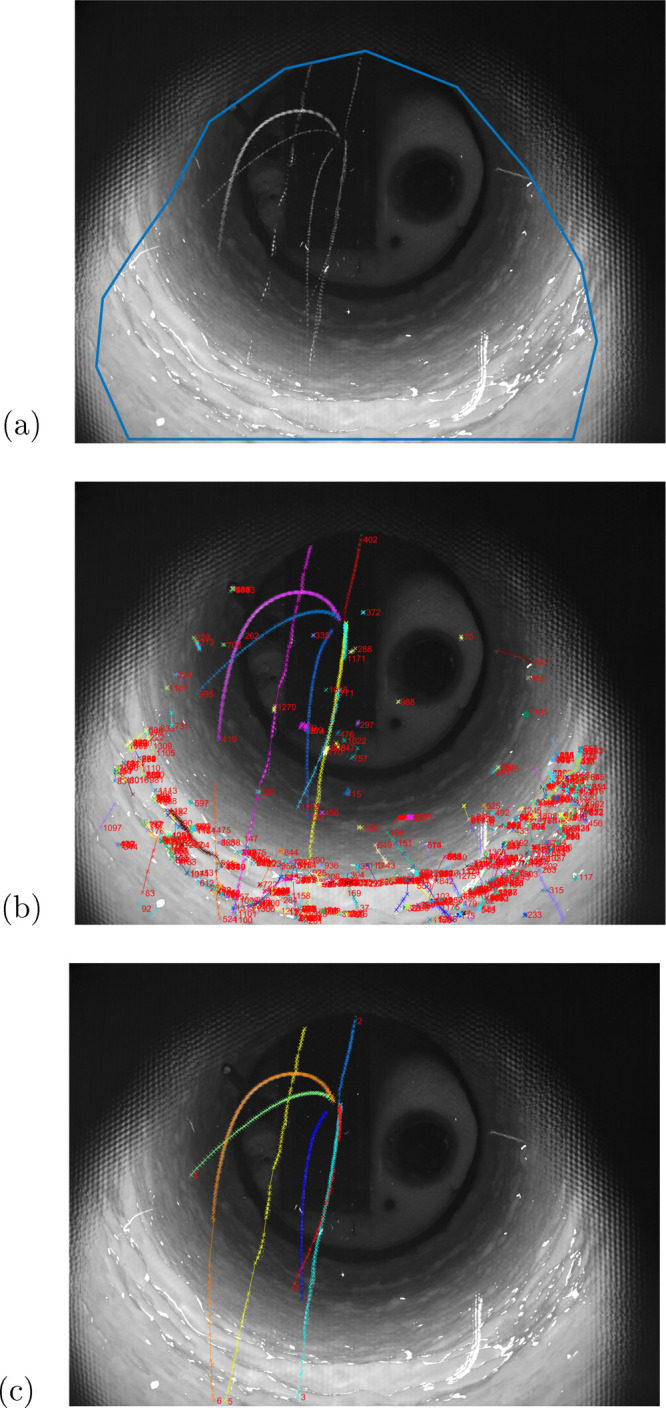
Example of optical ground truth and tracking outcome of wood chips. (a) Optical ground truth with an instance of defined region of interest (ROI) marked by a blue polygon. (b) 2D initial tracking result without postprocessing. (c) Postprocessing processed definitive 2D tracking result.

For the sake of a quantitative evaluation of the 2D tracking performance, we manually followed particles of each fraction within a certain length of frames and compared the thereby acquired ground truths with the tracking outcomes based on the measurements recommended in ref (29). Additionally, we also conduct an optical and qualitative evaluation by plotting the tracking trajectories on the sum of a set of adjacent difference images, which are obtained by subtracting the corresponding background image from each current image. The sum of the difference images serves as an optical ground truth that benefits a preliminarily visual assessment of the tracking performance. An optical ground truth is shown in Figure 10a. Withal, examples of tracking results of wood chips with and without postprocessing are depicted in Figure 10 as well.

As illustrated in Figure 10, the proposed postprocessing approach is able to optimize the tracking performance notably. To ensure the accuracy of the mentioned 3D polynomial regression under the condition that the particles must be conveyed through the lance, we ignore the trajectories that start away from the lance. The quantitative performance of the presented tracking algorithm is shown in Table 3, where α denotes the normalized paring score, β stands for the full normalized score, JSC is the Jaccard similarity index for positions, and JSC_θ_ is the Jaccard similarity index for tracks. The four measurements vary in the range of [0, 1], with 0 representing two entirely distinct sets of trajectories and 1 indicating two identical sets of trajectories with respect to a predefined deviation range.

**Table 3:** Evaluation of Tracking Performance with and without Post-Processing^a^

fraction/measure	α	β	JSC	JSC_θ_
wood chips	0.61	0.60	0.68	0.47
	0.67	0.67	0.76	1.0
confetti	0.05	0.05	0.06	0.22
	0.45	0.44	0.47	0.76
paper shreds	0.15	0.14	0.20	0.43
	0.60	0.59	0.61	0.84
PE granules	0.40	0.35	0.40	0.40
	0.62	0.59	0.64	0.84

a Upper values illustrate performance without post-processing, and lower values display performance with post-processing.

In light of the measurement values in Table 4, the postprocessing approach enhances the normalized paring score to an average of 0.6. Moreover, the Jaccard similarity for position is also increased to around 0.6, and the Jaccard similarity for tracks is raised to above 0.75. The most precise result occurs in tracking wood chips as a result of the concentrated mass and minor drag coefficient that lead to simple parabolic trajectories. On the contrary, confetti and paper shreds have relatively intricate motions, giving rise to their lower paring score and similarity.

**Table 4:** Space-Sliced Median Velocity of Each Fuel Fraction in the Depth Direction under Air Feed Pressures of 4.5 and 5 bar^a^

		wood chip		confetti		paper shred		PE granule	
air feed pressure	distance to lance	velocity	%	velocity	%	velocity	%	velocity	%
4.5 bar	0–1 m	13.51	100	7.41	90.00	5.20	63.11	8.61	100
	1–2 m	13.52	100	7.70	33.33	3.98	29.13	8.29	95.90
	2–3 m	12.92	82.72	4.67	8.33	4.38	4.83	9.59	44.10
	3–4 m	11.46	32.10		0		0	11.66	18.46
	4–5 m	9.14	6.17		0		0	9.01	3.59
	5–6 m		0		0		0		0
5 bar	0–1 m	12.21	100	7.66	90.70	5.58	75.00	8.49	100
	1–2 m	13.37	94.34	4.73	40.21	4.97	22.58	10.62	97.24
	2–3 m	12.18	62.26		0		0	10.68	80.69
	3–4 m	11.21	29.24		0		0	10.46	28.28
	4–5 m	10.91	7.55		0		0	9.03	6.21
	5–6 m		0		0		0	5.18	3.45

a The velocities are presented in 1 m distance intervals to the lance. The percentage value behind each velocity indicates the remaining percentage of the particle.

**Figure 11:**
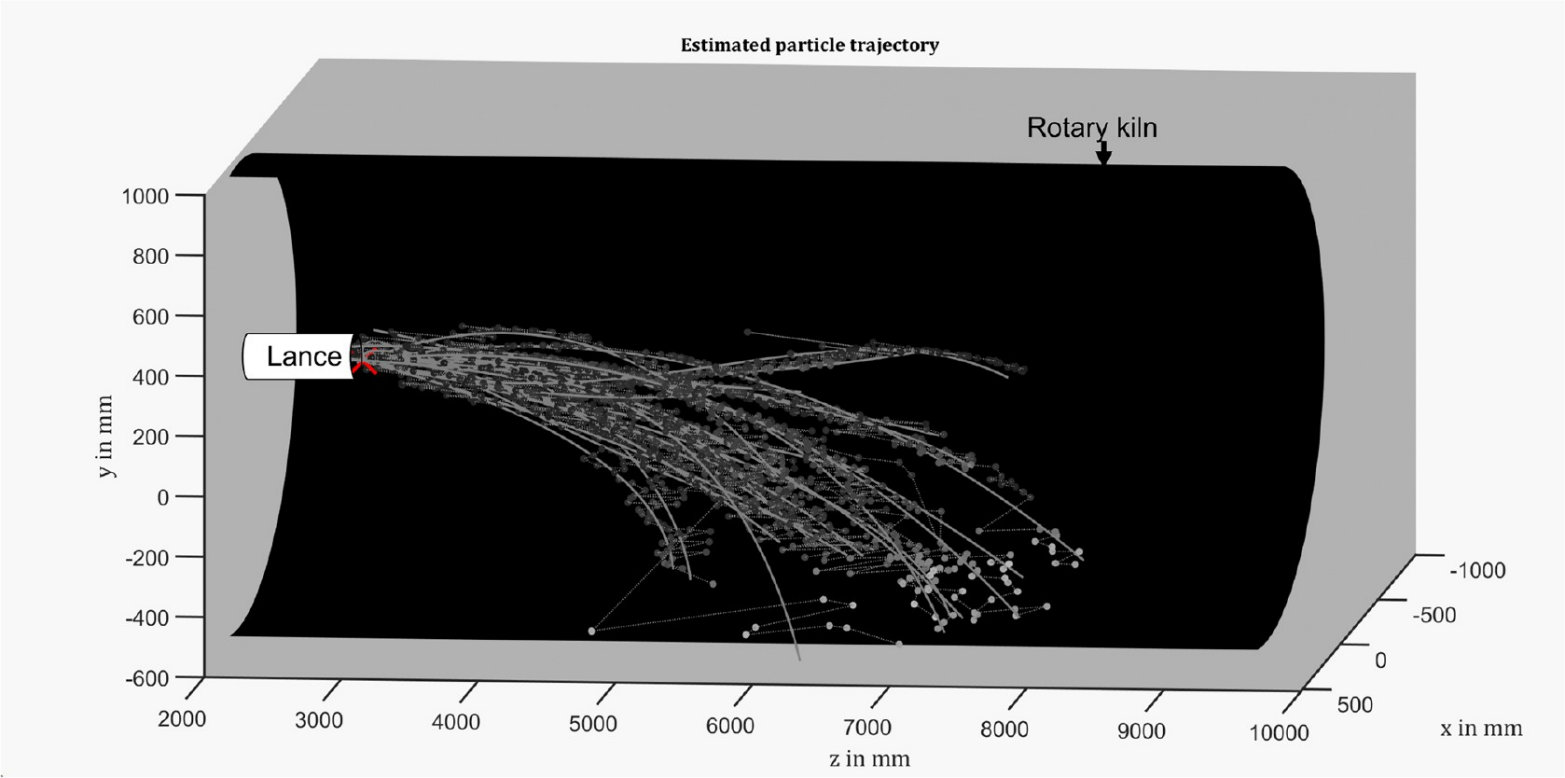
Estimated spatial trajectories with corresponding particle gray values. Each point represents the corresponding gray values of the detections.

The 2D trajectories are then converted into 3D particle trails that are estimated jointly in three spatial directions afterward. By virtue of the joint regression, the exact time point when a particle passes the lance can be computed, which is conducive to analyzing particles’ flight durations. An example of estimated particle trajectories is depicted in Figure 11, where the temporal particle positions are plotted in accordance with the corresponding average particle gray values.

## Discussion of Flight and Combustion Behaviors of RDF Particles

**Figure 12:**
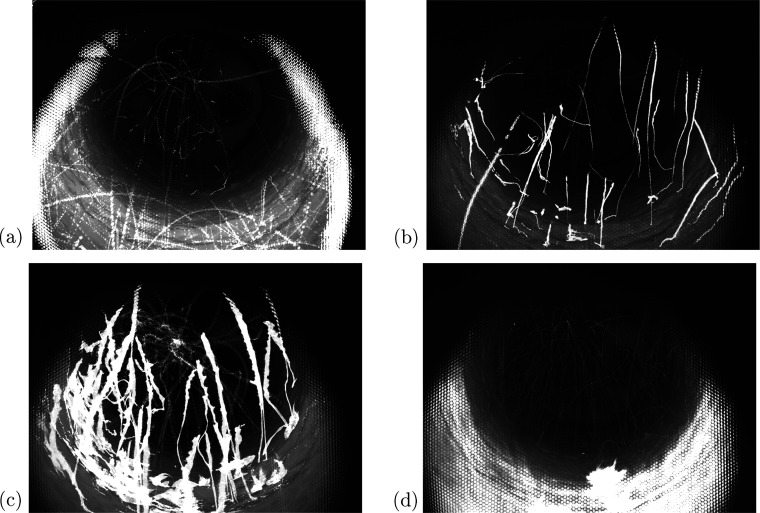
Examples of optical ground truth obtained by adding a set of adjacent difference images and a background image of each fraction. (a) Wood chips. (b) Confetti. (c) Paper shreds. (d) PE granules.

With the completion of multiple particle tracking-by-detection, investigations into a few particles’ flight and combustion behaviors are able to proceed based on the acquired spatial trajectories. In this section, we elaborate on the key findings of our study concerning flight durations of various RDF particles and their velocities in the depth direction under the condition of two distinct air feed pressures (4.5 and 5 bar). Withal, the ignition time of the particles has also been researched and analyzed. Before revealing the particles’ behaviors, we first present examples of the 2D optical ground truth of particles’ trajectories within a complete sequence of roughly 3000 frames in Figure 12 for an overview of their flight and combustion behaviors. As depicted in the figure, the trajectories of wood chips and PE granules are more regular compared to the traces of paper. Moreover, the paper materials are ignited during flight, whereas wood chips and PE granules do not combust during flight, basically.

### Flight Behaviors of RDF Particles

For the current work, we discuss two flight behaviors of the experimented upon four RDF fractions. At first, we cast light on the flight duration of the particles, which is also defined as the particles’ dwell time. Second, the RDF particles’ velocities in depth direction are presented. Actually, we are able to investigate velocities in three spatial directions. Notwithstanding, the horizontal and vertical velocities are of less interest since the horizontal velocity can be relatively random, and the vertical motion approximates free fall. Therefore, we only illustrate the velocity in depth.

#### Flight Duration (Dwell Time)

**Figure 13:**
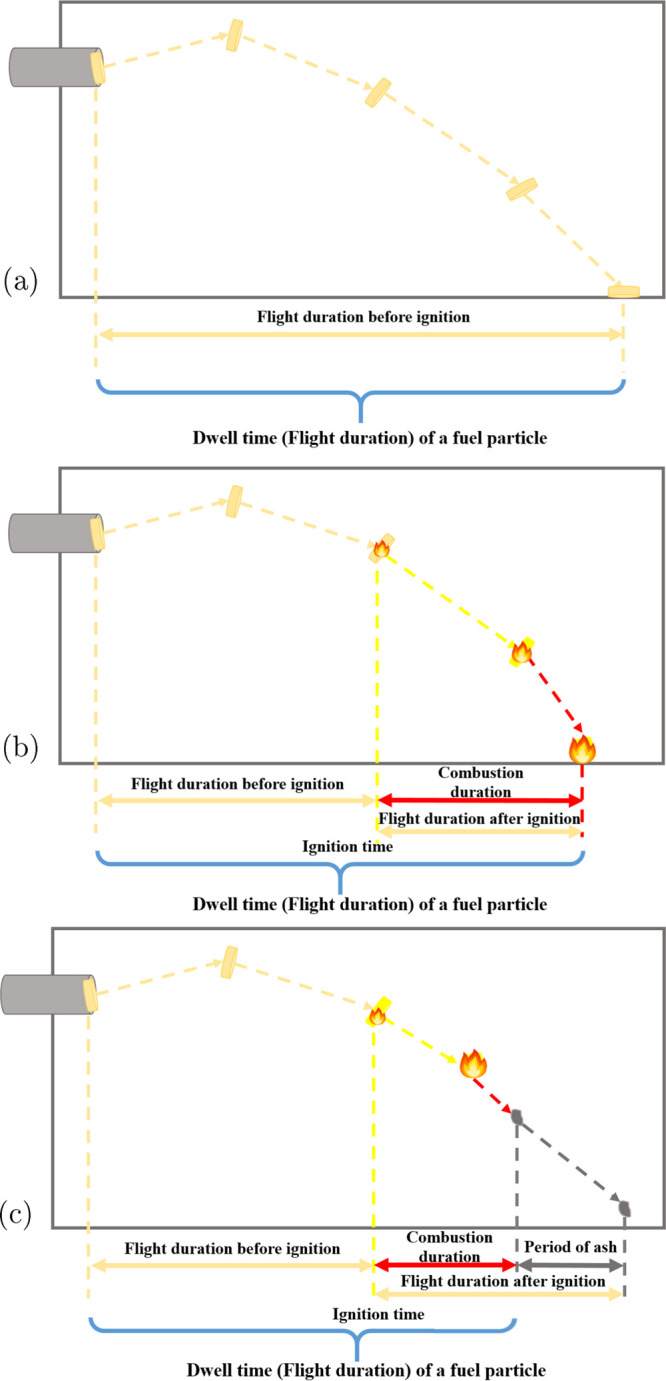
Definitions of the dwell time (flight duration) in different situations. (a) Dwell time of nonburning particles. (b) Dwell time of burning particles that do not burn out during the flights. (c) Dwell time of burning out particles.

First of all, we would like to highlight the flight duration defined in the present work. The flight duration of a particular particle refers to the time interval that starts from passing the lance and ends by landing or burning out. In our work, we treat the flight duration and the dwell time as the same concept. In Figure 13, three general cases are depicted. Figure 13a presents the flight duration of a nonburning particle. For a burning particle, if the particle lands before it burns out, we count the time until landing as the flight duration, as delineated in Figure 13b. In contrast, if the particle ignites rapidly and vanishes already before landing, the flight duration ends at burning out, as shown in Figure 13c.

**Figure 14:**
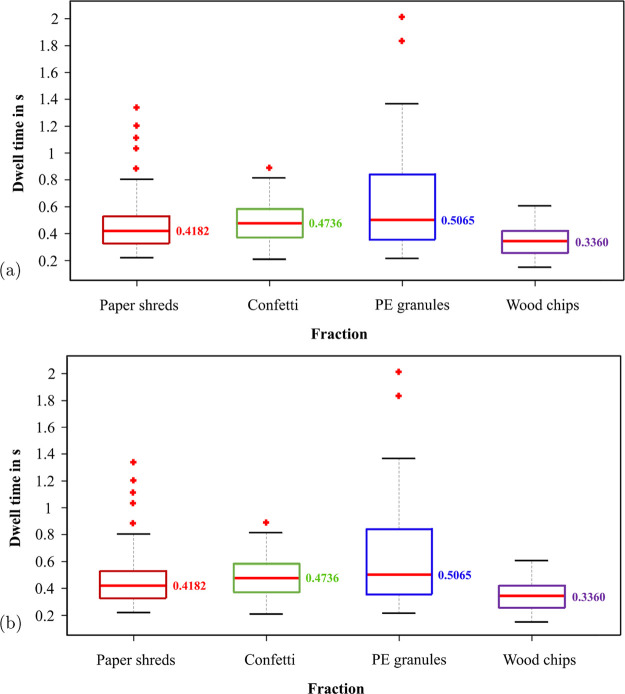
Box plot of the dwell times of various RDF fractions. (a) Dwell times of the fractions at 4.5 bar of air feed pressure. (b) Dwell times of the fractions at 5 bar of air feed pressure.

In Figure 14, the flight durations of the RDF fractions are represented by box plots. The red line within each box represents the median flight duration of the corresponding fraction, and the bottom and top of the box indicate the 25th and 75th percentiles, respectively. The whiskers extend to the extreme values that are not considered outliers, while the outliers are shown with the symbol “+”.

The dwell time at 4.5 bar of air feed pressure is illustrated in Figure 14a. As depicted in the figure, the average flight duration of each fraction is approximately between 0.3 and 0.5 s. Among them, PE granules have the longest and most unevenly distributed flight time, while wood chips have the shortest and most concentrated distribution on average. The flight durations of paper shreds and confetti are very similar in terms of median and distribution. As also indicated in Figure 14a, a few outliers with a flight duration of almost 1 s can also be found, which can be attributed to occasionally occurring complex trajectories of the particles.

Figure 14b shows the flight durations of individual fractions at 5 bar of feed pressure. Overall, the dwell times at 5 bar do not differ significantly from the previous values at 4.5 bar, except for PE granules, whose average dwell time is 0.12 s shorter. In general, the median flight durations of all fractions are between 0.3 and 0.4 s. Confetti has the longest flight duration, while wood chips have the shortest dwell time due to their rapid movements. As a consequence of the irregular motion of paper, their flight durations’ distributions are with high variance.

#### Velocity in Depth Direction

**Figure 15:**
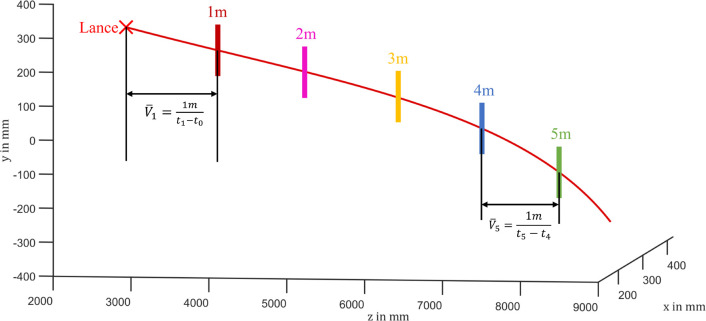
Illustration of the space-sliced velocity in the depth direction. The lance is marked by a red cross.

The airflow field inside the rotary kiln results in the dependency of the velocity in depth on the particle’s flight distance. Therefore, as schematically illustrated in Figure 15, we report the statistical space-sliced velocity with a distance interval of 1 m starting from the lance. The space-sliced velocity of a particular particle can be mathematically interpreted as
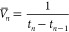
1where *t*_*n*_ denotes the time when the estimated particle trajectory passes the *n* meter distance and *t*_*n*–1_ is the time when the estimated particle trajectory passes the *n* – 1 m distance. Therefore, *V̅*_*n*_ stands for the mean velocity of *n* – 1 to *n* meters. In the following, the velocities of the RDF fuel particles are presented separately according to their fractions. We display the velocities in Table 4, where the velocity columns indicate the median velocities with the corresponding distances to the lance in the depth direction. In addition, we also present the percentage of remaining particles at each distance interval behind the median velocities.

##### Wood Chip

The third and fourth columns of Table 4 show the space-sliced depth velocity of wood chips at 4.5 and 5 bar of air feed pressure. In the experiment with 4.5 bar of feed pressure, the median of the initial velocity of the wood chips can reach 13.5 m/s and then slowly decreases to about 9 m/s. When the feed pressure is increased to 5 bar, the median depth velocity of the particles as they exit the lance decreases, which then rises slightly and decreases again afterward. It should be noted that the particles do not really speed up. The enhancement of the median velocity is the consequence of the slow particles that have already landed early. In general, the velocity at 5 bar of feed pressure differs insignificantly from that at 4.5 bar. With respect to the percentage of wood chips remaining at each distance, all wood chip particles can move beyond 1 m in depth, and the majority can reach more than 3 m at both air feed pressures. Approximately 30% of wood chips can move up to a distance of 4 m from the lance, and only very few can reach a distance of more than 5 m.

##### Confetti

The fifth and sixth columns of Table 4 illustrate the space-sliced depth velocity of confetti at 4.5 and 5 bar of air feed pressure. As shown in the table, the median velocity can reach about 7.5 m/s within a distance of 2 m from the lance at 4.5 bar. As a consequence of the early ignition of the confetti, the particles do not penetrate far in the depth direction. Therefore, hardly any confetti particles are discovered beyond a distance of 3 m. When the feed pressure is raised to 5 bar, the speed of the confetti differs slightly. Moreover, the particle velocity decreases significantly with increasing distance. Concerning the percentage of confetti remaining at each distance, beyond 90% of the confetti can move to 1 m from the lance, whereas less than half of the particles are not able to reach a distance of 2 m.

##### Paper Shreds

The seventh and eighth columns of Table 4 show the space-sliced depth velocity of paper shreds. As shown in the upper part of the table, the paper shreds present slower movements in the depth direction and, likewise, shorter depth motions. With a distance of 1 m from the lance, the medial velocity is 5.2 m/s, and then the velocity decreases with increasing distance. The amount of remaining particles at a distance of more than 2 m decreases sharply. At distances greater than 3 m, no particles are detected. Due to the fast transverse motion of the paper shreds, many paper shreds touch the side walls before moving far from the lance. When the feed pressure is increased to 5 bar, the depth velocity of the paper shreds increases as well. Nevertheless, the initial velocity is significantly influenced by the speed of the conveying. The velocity decreases rapidly with a distance of beyond 1 m from the lance. A sufficient number of particles with more than 2 m motions from the lance are not discovered for statistical evaluation. As for the percentage of paper shreds remaining at each distance, similar to confetti, the paper shreds do not move far in the depth direction. Around 60% of the paper shreds reach a depth distance of 1 m at 4.5 bar of feed pressure, while 75% of the paper shreds move to a distance of 1 m at 5 bar. At both feed pressures, less than 30% of the particles indicate a motion of over 2 m in the depth direction.

##### PE Granule

The last two columns show the space-sliced depth velocity of PE granules at 4.5 and 5 bar of air feed pressure. Compared to the first two paper fractions, PE granules are significantly faster and move further in the depth direction. Under a feed pressure of 4.5 bar, the median velocity of PE granules reaches approximately 8 m/s for the first 2 m and then slowly increases. Since extremely slow particles are not able to penetrate further than 2 m in the depth direction and exist therefore only in a short distance, the velocity increases with a distance beyond 2 m. This can also be proved by the percentage of remaining particles on the bottom. While over 95% of particles are capable of reaching 2 m in the depth direction, less than 45% of PE granules can move up to 3 m. From a distance of 5 m, remaining PE particles are hardly discovered. When the feed pressure is increased to 5 bar, the initial velocity of PE granules decreases slightly. From a distance of 1 m, the velocity gradually increases to around 10 m/s. Over a 4 m distance, the median velocity decreases lightly. Several PE granule particles can also be detected at a distance of more than 5 m. Nevertheless, their median velocity decreases considerably. At both air feed pressures, all PE granules can move more than 1 m in the depth direction, and the vast majority of PE granules can exceed 2 m. With a feed pressure of 5 bar, the percentage of remaining PE granules with a motion of beyond 2 m reaches 80%. At a distance of more than 4 m, the remaining percentage of PE granules is significantly lower.

### Combustion Behaviors of RDF Particles

As indicated in Figure 12, wood chips and PE granules could not be ignited by the heat in the rotary kiln during flight. Therefore, we only delve into the combustion behaviors of confetti and paper shreds hereafter. The ignition times are shown in 0.1 s intervals in Table 5.

**Table 5:** Ignition Time of Confetti and Paper Shreds under Air Feed Pressures of 4.5 and 5 bar^a^

air feed pressure		<0.1 s	0.1–0.2 s	0.2–0.3 s	0.3–0.4 s	0.4–0.5 s	>0.5 s	no ignition
4.5 bar	confetti	3%	10%	2%	6%	2%	3%	74%
	paper shred	21%	16%	10%	18%	5%	1%	29%
4.5 bar	confetti	0%	4%	9%	4%	0%	2%	81%
	paper shred	6%	14%	6%	9%	2%	5%	58%

a The ignition time is divided into 0.1 s intervals, with the percentage value indicating the percentage of particles ignited within the corresponding time interval.

#### Confetti

Table 5 illustrates the ignition time of confetti schematically at 4.5 and 5 bar of air feed pressure. Under a feed pressure of 4.5 bar, only 26% of the confetti particles ignite. Among them, only 3% of the confetti ignites within 0.1 s. Most of the burning confetti combusts between 0.1 and 0.2 s. The percentage of burning particles decreases as the feed pressure increases. With a feed pressure of 5 bar, only less than 20% of confetti ignites, and no confetti ignites within 0.1 s after leaving the lance. The majority of burning confetti ignites between 0.2 and 0.3 s.

#### Paper Shreds

Table 5 shows the ignition time of paper shreds at 4.5 and 5 bar of air feed pressure. Compared to the confetti, the paper shreds have an earlier ignition time. At 4.5 bar of feed pressure, more than 70% of the paper shreds are ignited in flight, and over 20% combust within 0.1 s. The vast majority of paper shreds burn within 0.4 s. At 5 bar of feed pressure, only around 40% of the paper shreds ignite. Furthermore, the ignition occurs later than at 4.5 bar on average, and only 6% of the paper shreds are ignited within 0.1 s. The proportion of paper shreds that ignite in more than 0.5 s is also higher than at 4.5 bar.

## Conclusion and Outlook

In the paper, we elaborate on a novel plenoptic camera based measurement system to investigate various RDF fractions’ flight and combustion behaviors by processing captured images. The applied image processing based on the concept of tracking-by-detection contains a novel combined detection approach and a 2.5D multiple particle tracking method with a postprocessing framework. The proposed detection approach synthesizes the detections of a 2D gray value based algorithm SIFT and a 3D clustering method DBSCAN and is proven to optimize the detection performance. Based on the detection outcomes, multiple particles are able to be tracked using the linear Kalman filter and 2.5D GNN. To tackle the problems that occurred in the tracking results, for instance, faulty tracklets or incomplete tracklets, we developed a postprocessing framework, which modifies and connects tracklets by taking full advantage of the motion similarity between obtained tracklets. The effectiveness of the detection approach and the postprocessing framework are quantitatively assessed by manually labeled detection ground truth and tracking ground truth. The outcomes of the image processing are complete 2D particle trajectories that are converted into 3D in accordance with the particle spatial coordinates provided by the plenoptic camera afterward. To compensate for the fluctuations of the converted spatial trajectories, we estimate them with polynomials under the condition that all particles must be conveyed through the lance. Subsequently, we investigate the flight and combustion behaviors of the fuel particles utilizing the polynomial trajectories. The studied flight behavior consists of the dwell time (flight duration) of the fuel fractions and their space-sliced velocity in the depth direction. To explore the combustion behavior, we report the ignition times of various fuel fractions. The obtained fuel properties are compared with a CFD model, which provides similar statements to the experimental outcomes.^30^ Thereby, the reliability and availability of the proposed novel measurement system can be proved.

Our experiments with various fuel particles indicate the adequacy of the proposed measurement system for research of RDF flight and combustion properties. Future research will aim at investigating the robustness of the presented methods, namely, to experiment with them on other measurements with distinct fuel fractions or experimental setups.
